# A stomatal safety-efficiency trade-off constrains responses to leaf dehydration

**DOI:** 10.1038/s41467-019-11006-1

**Published:** 2019-07-30

**Authors:** Christian Henry, Grace P. John, Ruihua Pan, Megan K. Bartlett, Leila R. Fletcher, Christine Scoffoni, Lawren Sack

**Affiliations:** 1 Department of Ecology and Evolutionary Biology, University of California Los Angeles, 621 Charles E. Young Drive South, Los Angeles, CA 90095 USA; 2 Department of Integrative Biology, University of Texas at Austin, 2415 Speedway, Austin, TX 78712 USA; 3 School of Ecology and Environment, Inner Mongolia University, 235 University West Road, 010021 Hohhot, Inner Mongolia China; 4 Department of Viticulture and Enology, University of California Davis, One Shields Avenue, Davis, CA 95616 USA; 5 Department of Biological Sciences, California State University, Los Angeles, 5151 State University Drive, Los Angeles, CA 90032 USA

**Keywords:** Plant physiology, Ecophysiology

## Abstract

Stomata, the microvalves on leaf surfaces, exert major influences across scales, from plant growth and productivity to global carbon and water cycling. Stomatal opening enables leaf photosynthesis, and plant growth and water use, whereas plant survival of drought depends on stomatal closure. Here we report that stomatal function is constrained by a safety-efficiency trade-off, such that species with greater stomatal conductance under high water availability (*g*_max_) show greater sensitivity to closure during leaf dehydration, i.e., a higher leaf water potential at which stomatal conductance is reduced by 50% (Ψ_gs50_). The *g*_max_ - Ψ_gs50_ trade-off and its mechanistic basis is supported by experiments on leaves of California woody species, and in analyses of previous studies of the responses of diverse flowering plant species around the world. Linking the two fundamental key roles of stomata—the enabling of gas exchange, and the first defense against drought—this trade-off constrains the rates of water use and the drought sensitivity of leaves, with potential impacts on ecosystems.

## Abstract (web summary)

Stomata enable gas exchange for photosynthesis but close to promote survival during drought. Here, Henry et al. provide evidence for a safety-efficiency trade-off whereby plants with greater stomatal conductance under well-watered conditions are more sensitive to stomatal closure during dehydration.

## Introduction

Stomata exert major influences on plant and ecosystem productivity and drought tolerance^1–3^. Across the diversity of plant species, leaves with a greater area of open stomatal pores have higher stomatal conductance (*g*_s_), and typically, greater rates of photosynthetic CO_2_ assimilation and of transpiratory water loss^4–6^. However, plants must maintain their hydration within narrow limits, and a high *g*_s_ and transpiration rate drive declines in water potential throughout the plant^7^, which would cause mesophyll damage and xylem embolism during drought^8^. Plants thus close stomata in response to decreasing leaf water potential (Ψ_leaf_). The decline of *g*_s_ (i.e., stomatal closure) with decreasing Ψ_leaf_ is important among the complex of internal and external factors that determine overall stomatal responses, including root-derived signals, ambient irradiance and CO_2_^9–11^ and influences the dynamics of gas exchange and productivity and drought tolerance across plant species^1,12–15^.

**Fig. 1:**
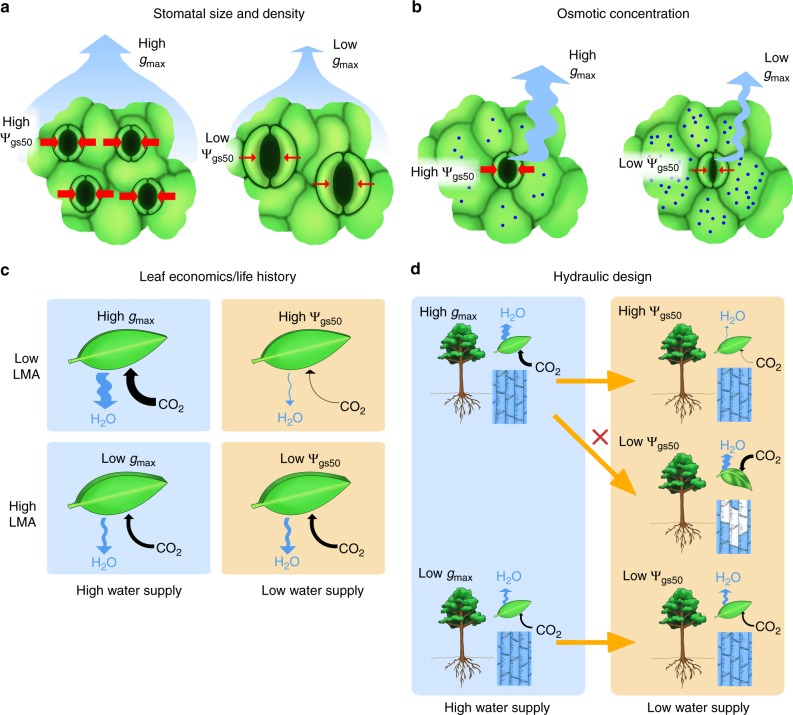
Hypothesized rationales for a stomatal safety-efficiency trade-off. **a** Stomatal size and density: leaves with smaller, denser stomata (left) have higher maximum stomatal conductance (*g*_max_), and stomata more sensitive to closure during drought (i.e., higher Ψ_gs50_, indicated by thicker red lines) than leaves with larger, less dense stomata (right). **b** Osmotic concentration: Leaves with weaker cellular osmotic concentrations (i.e., higher osmotic potentials) at full turgor and turgor loss (left) are associated with higher *g*_max_ and higher Ψ_gs50_ than leaves with stronger osmotic concentrations (i.e., lower osmotic potentials) (right). **c** Leaf economics and life history trade-off: Species selected for greater resource acquisitiveness, and with lower leaf mass per area (LMA; top row) would have higher *g*_max_ and photosynthetic rate under high water supply (left column), and more sensitive stomatal closure under low water supply (right column) than species with high LMA (bottom row), which have lower *g*_max_ and photosynthetic rate under high water supply, and can better maintain stomatal conductance and photosynthetic rate under low water supply. **d** Plant hydraulic design: Under high water supply (left column), species with high *g*_max_ have higher photosynthetic rate than species with low *g*_max_ and both maintain leaf turgor and xylem water column continuity; under low water supply (right column), species with high *g*_max_ must show sensitive stomatal closure (i.e., higher Ψ_gs50_) and therefore strong reduction of photosynthetic rate to avoid leaf damage and xylem embolism (right column, top two schematics), whereas species with low *g*_max_ can maintain stomatal conductance and photosynthetic rate under low water availability (right column, lowest schematic)

One potentially general constraint on the response of *g*_s_ to Ψ_leaf_ would be a trade-off between high maximum stomatal conductance (*g*_max_) in hydrated leaves and greater sensitivity to closure during dehydration, i.e., a higher Ψ_leaf_ at 50% loss of stomatal conductance (Ψ_gs50_). Such trade-offs between “safety” and “efficiency”, or, similar in logic, between “stress tolerance” and “potential growth” are common in plant and animal biology^16,17^ and industrial systems^18^. A well-known hypothesis in whole plant physiology is a constraint on internal water transport known as the hydraulic safety-efficiency trade-off: an association across species between high values for the maximum stem or leaf hydraulic conductivity and a greater sensitivity to decline during dehydration^19–21^. Hydraulic safety-efficiency trade-offs are often strong within lineages of closely related species, significant though weak across the sampled diversity of species globally^19,20^, and may contribute to adaptation to habitat and climate^22,23^. The evolution of an analogous *g*_max_–Ψ_gs50_ trade-off would be expected based on multiple, nonexclusive rationales from stomatal biomechanics, hydraulic design, and life history theory (Fig. 1a–d).

First, a *g*_max_−Ψ_gs50_ trade-off might arise mechanistically according to variation in stomatal size and density (Fig. 1a). Smaller, denser stomata are associated with higher *g*_max_, both by contributing to a greater anatomical maximum stomatal conductance (*g*_max,anatomy_) and to a greater stomatal opening ratio during gas exchange (*g*_max ratio_ = *g*_max_/*g*_max,anatomy_)^5,24,25^. Further, smaller stomata have a greater surface area to volume ratio, facilitating ion exchange and thus stronger and faster movements in response to changing irradiance and leaf hydration status^26–28^.

Additionally, a *g*_max_–Ψ_gs50_ trade-off might arise due to variation in solute concentrations within leaf cells (Fig. 1b). For stomata to open, guard cells must accumulate solutes from the apoplast, driving water uptake to build sufficient hydrostatic pressure to inflate against the surrounding pressure of the epidermal pavement cells^27,29^. Opening to a higher *g*_max_ may thus be mechanically facilitated, requiring less guard cell ion uptake, when epidermal pavement cells have lower osmotic concentration and lower turgor pressure at full hydration^27,29^, which tissue-scale studies have shown would be associated with a higher bulk leaf osmotic potential at full turgor (*π*_o_)^30^. A higher *π*_o_ would also cause greater stomatal sensitivity to closure under drought, as it corresponds to a higher turgor loss point (*π*_tlp_), i.e., greater sensitivity to wilting, and stomatal closure is a typical wilting response^12,31–34^.

A *g*_max_−Ψ_gs50_ trade-off may also arise as a leaf economic or life history trade-off (Fig. 1c). Theoretical and empirical analyses support trade-offs across species among traits that confer benefits for resource acquisition and those that confer stress tolerance^35,36^. Species adapted to high resource supplies tend to allocate less to leaf structural protection, resulting in lower leaf mass per area (LMA) and higher rates of photosynthesis per unit leaf mass, at the cost of stronger photosynthetic declines under resource scarcity, and shorter leaf lifetimes. By contrast, species adapted to low resource supplies tend to invest in structural protection and higher LMA at the expense of photosynthetic machinery, and to maintain leaves longer into periods of scarcity and to achieve longer leaf lifetimes^36,37^. A *g*_max_–Ψ_gs50_ trade-off would be consistent with leaf economic and life history trade-offs, such as between maximum photosynthetic rate under well-watered conditions, and sensitivity to photosynthetic decline during drought^36^, given that *g*_max_ is a key determinant of maximum photosynthetic rate^6^, and Ψ_gs50_ of the ability to maintain photosynthesis during drought^1^.

Our fourth and final hypothesis was that a *g*_max_–Ψ_gs50_ trade-off may balance photosynthetic productivity against protection from dehydration stress under atmospheric or soil drought (Fig. 1d). A higher *g*_max_ would facilitate rapid photosynthetic rates in moist soil, but would also result in greater transpiration rates and steeper declines in water potential throughout the plant, which under drought would increase the danger of xylem embolism^1^. Species with higher *g*_max_ thus would require greater sensitivity to closure to avoid dehydration stress during soil and/or atmospheric drought. The *g*_max_–Ψ_gs50_ trade-off would enable plants to maintain high photosynthetic rates under high water availability, yet minimize dehydration stress during drought.

**Fig. 2:**
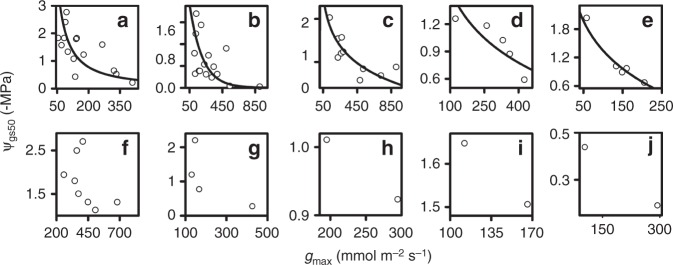
The generality of the stomatal safety-efficiency trade-off. Relationship of maximum stomatal conductance (*g*_max_) and sensitivity to stomatal closure (Ψ_gs50_) for (**a**) 15 California species grown in a common garden design for this study (*r* = 0.69; *P* = 0.005; phylogenetic least squares regression), and in analyses of previous studies of stomatal responses in excised leaves or dehydrating plants of diverse species, measured with different techniques, and under different growing conditions (Pearson correlations): (**b**) 16 diverse angiosperm species (*r* = 0.50; *P* = 0.05)^74^, (**c**) 10 Chinese *Ficus* species^32^ (*r* = 0.82; *P* = 0.003), (**d**) five European tree species^33^ (*r* = 0.79; *P* = 0.03), (**e**) five tree species^75^ (*r* = 0.95; *P* = 0.009), (**f**) eight tree species of Costa Rican dry forest^76^ (*r* = 0.55; *P* *>* 0.05), (**g**) four woody species^46^, (**h**) two *Vitis vinifera* cultivars^77^, (**i**) two *Vaccinium* species of subalpine Austria^78^, and (**j**) two varieties of a fern species^64^. The *g*_max_ and Ψ_gs50_ values were derived from fitted curves (Supplementary Fig. 1). Lines are standard major axes for log-transformed data, i.e., for power-law fits. Different scales were used in the panels to highlight the generality of the trend across studies of species diverse in stomatal responses to leaf water status. Source data are provided as a Source Data file

We report on the demonstration of *g*_max_–Ψ_gs50_ trade-off and its mechanistic basis in a controlled experiment on 15 California tree and shrub species, and in analyses of a unique compiled database of previous studies of stomatal responses for diverse species (Fig. 2; Supplementary Data 1–8).

## Results

### Relationship between *g*_max_ and Ψ_gs50_

Across the California species, *g*_max_ varied by seven-fold, and Ψ_leaf_ at 20%, 50%, and 80% stomatal closure (Ψ_gs20_, Ψ_gs50_, and Ψ_gs80_) by 2.1, 2.6, and 3.1 MPa, respectively (stomatal parameters determined from fitted curves; Fig. 2a; Supplementary Fig. 1; Supplementary Data 1, 3, and 4). Across these 15 species, a higher *g*_max_ was correlated with higher values of Ψ_gs20_, Ψ_gs50_, and Ψ_gs80_ (Fig. 2a; Supplementary Data 5 and 6). Likewise, our analyses of the data from nine previous studies of stomatal responses in datasets on diverse species of mainly woody angiosperms (Supplementary Data 2) showed in each case an empirical tendency for species with high *g*_max_ to have higher values of Ψ_gs20_, Ψ_gs50_, and Ψ_gs80_ (Fig. 2b–j; Supplementary Data 7). Five of the six studies that tested ≥5 species showed a significant *g*_max_–Ψ_gs50_ trade-off (Fig. 2a–f), with the slope of the relationship varying significantly across studies (Supplementary Data 2).

**Fig. 3:**
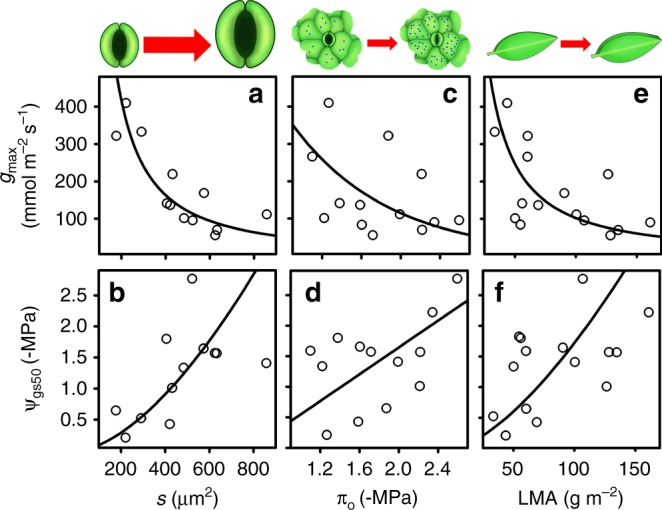
Testing hypothesized rationales for the stomatal safety-efficiency trade-off. Relationships of maximum stomatal conductance (*g*_max_) and the leaf water potential at 50% stomatal closure (Ψ_gs50_) with (**a**) and (**b**) stomatal size (*s*; *n* = 12 species for which data were available); (**c**) and (**d**) osmotic potential at full turgor (*π*_o_; *n* = 13 species for which data were available); and (**e**) and (**f**) leaf mass per area (LMA; *n* = 15 species). Lines are standard major axes for untransformed or log-transformed data, i.e., for linear or power-law fits, depending on which showed a stronger fit. Phylogenetic least squares regression *r* values for panels (**a**)–(**f**), respectively, were −0.56, 0.61, −0.82, 0.73, −0.61, and 0.56 (*P* = 0.0009–0.04). The *g*_max_ and Ψ_gs50_ values were derived from fitted curves (Supplementary Fig. 1). Source data are provided as a Source Data file

**Fig. 4:**
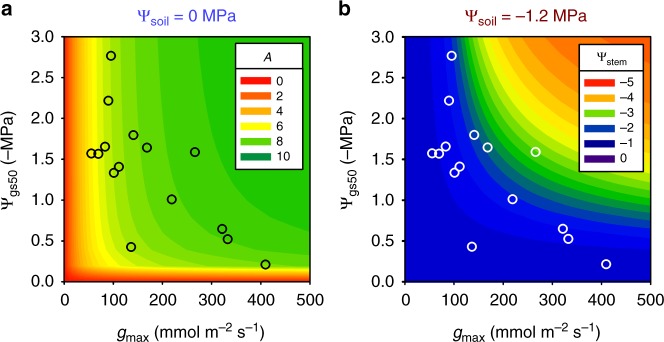
Testing for a benefit in plant hydraulic design of a stomatal efficiency-safety trade-off. **a** contour plot shows modeled light saturated photosynthetic rate (*A*; units μmol m^−2^ s^−1^) for simulated species with combinations of maximum stomatal conductance (*g*_max_) and sensitivity to stomatal closure (Ψ_gs50_) under high water availability, i.e., soil water (Ψ_soil_) of 0 MPa; species with higher *g*_max_ had higher *A*, whereas species with sensitive stomatal closure, i.e., higher (lower numerical values of) Ψ_gs50_ had reduced *A.* **b** Modeled stem water potential (Ψ_stem_; units: MPa) for these simulated species under low water availability, i.e., Ψ_soil_ of −1.2 MPa; species with higher *g*_max_ had steeper declines in Ψ_stem_, whereas species with higher (lower numerical values of) Ψ_gs50_ were protected from dehydration stress. The *g*_max_ vs. Ψ_gs50_ trade-off for the 15 California species positioned these species in the optimal zone, with high enough *g*_max_ to achieve moderate to high values of *A* under high water availability, and sensitive enough stomatal closure (i.e., low enough numerical values of Ψ_gs50_) to avoid hydraulic damage during drought. See “Methods” section for model description and parameterization, and Supplementary Fig. 3 for additional simulations, including of impacts on leaf water potential. Source data are provided as a Source Data file

We tested four putative mechanisms for the *g*_max_–Ψ_gs50_ trade-off in the California species, and found support for each (Figs. 3 and 4). First, we tested whether the *g*_max_–Ψ_gs50_ trade-off might arise mechanistically according to variation in stomatal size and density (Fig. 1a). The California species varied strongly in stomatal density, size, *g*_max,anatomy_, and *g*_max ratio_ (Supplementary Data 1). As hypothesized, leaves with smaller stomata had greater *g*_max_ and higher values of Ψ_gs50_ and Ψ_gs80_, and these were associated with higher *g*_max ratio_, rather than with higher *g*_max,anatomy_ (Fig. 3a, b; Supplementary Fig. 2; Supplementary Data 5 and 6).

Second, we tested whether the *g*_max_−Ψ_gs50_ trade-off might arise due to variation in solute concentrations within leaf cells (Fig. 1b). The California woody species varied strongly in *π*_o_ and *π*_tlp_ (Supplementary Data 1), and all species began closing stomata with dehydration well before *π*_tlp_, at which point many had reduced stomatal conductance by >80% (Supplementary Fig. 1). Species with lower *π*_o_ had lower *g*_max_ and species with lower *π*_o_ and *π*_tlp_ had lower Ψ_gs50_ and Ψ_gs80_ values (Fig. 3c, d; Supplementary Data 5 and 6).

Third, we tested whether the *g*_max_−Ψ_gs50_ trade-off might arise as a leaf economic or life history trade-off linked with leaf mass per unit area (LMA; Fig. 1c). Across the California species, *g*_max_ and Ψ_gs50_ were both negatively related to *LMA* (Fig. 3e, f; Supplementary Data 5 and 6).

Finally, we tested whether a *g*_max_−Ψ_gs50_ trade-off may balance photosynthetic productivity against protection from dehydration stress under atmospheric or soil drought (Fig. 1d). Using a plant hydraulic-stomatal-photosynthetic model^21,38^, we simulated species with typical physiological parameters and differing in only their *g*_s_ versus Ψ_leaf_ responses, i.e., in their *g*_max_ and Ψ_gs50_ values, and calculated their light-saturated photosynthetic rates (*A*), and leaf and stem water potentials under high water availability, i.e., soil water potential (Ψ_soil_) of 0 MPa, and soil drought, i.e., Ψ_soil_ of −1.2 MPa. Species with high *g*_max_ had higher *A* irrespective of Ψ_soil_ (Fig. 4a; Supplementary Fig. 3A–D), but showed steep declines in both leaf and stem water potentials under low Ψ_soil_ (Fig. 4b; Supplementary Fig. 3B, C, E and F). Conversely, species with sensitive stomatal closure (i.e., high Ψ_gs50_) showed lower *A* under high water availability due to partial stomatal closure during transpiration, but substantially less leaf and stem dehydration stress under drought (Fig. 4b; Supplementary Fig. 3). The *g*_max_−Ψ_gs50_ trade-off would thus enable the California species to avoid low *A* under high water availability, as well as hydraulic damage during drought (Fig. 4 and Supplementary Fig. 3).

## Discussion

A strong generality was observed for the *g*_max_−Ψ_gs50_ trade-off across woody angiosperm species. Further study is required to test for this trade-off across a yet broader phylogenetic sample of angiosperms, such as herbs, including grasses, and other major lineages, such as ferns and gymnosperms, which also close stomata in response to declining Ψ_leaf_, but differ in aspects of stomatal control physiology^39,40^. In the single study amenable to re-analysis of the response of *g*_s_ to Ψ_leaf_ for a fern species, the comparison of two varieties was consistent with the trade-off (Fig. 2j).

We found support for multiple mechanisms as putative causes of the *g*_max_−Ψ_gs50_ trade-off^20,38^. Thus, the trade-off could be explained by species with smaller, denser stomata also having more sensitive closure. Indeed, the more sensitive stomatal closure of species with small stomata is consistent with their tendency to respond more quickly and/or strongly to transitions in light, water status, and VPD, conferring greater tolerance of a variety of stresses^25,26,28,40^. The trade-off could also be explained by species with lower bulk leaf osmotic potentials having lower *g*_max_ and also less sensitive closure. While the contribution of low *π*_o_ and *π*_tlp_ to the maintenance of gas exchange during drought has been well recognized^12^, our findings indicate a major cost, explaining why these traits are not universal, as the association of low *π*_o_ with low *g*_max_ would restrict gas exchange under high water availability. The trade-off was also consistent with life history theory, given that low LMA species with higher *g*_max_ would be expected to show greater stress sensitivity. Finally, the trade-off is consistent with theory that stomatal conductance and its dynamics evolved to enable maximum water use and therefore photosynthetic productivity while reducing risk of hydraulic failure^1,8,20^. The mechanisms proposed would be interactive and mutually reinforcing, though our results do not exclude further mechanisms for the trade-off, including a role for the hormone abscisic acid, which is involved in stomatal behavior^41^.

The *g*_max_−Ψ_gs50_ trade-off provides an important constraint on stomatal behavior in response to water supply. Stomatal behavior in response to soil and atmospheric water supply has often been considered according to a dichotomy or continuum from “isohydric” to “anisohydric” behaviors. Isohydric plants maintain high organ water potentials by closing stomata early during a drought, whereas anisohydric plants tolerate low water potentials, and maintain open stomata for prolonged photosynthesis^11^. However, recent work has shown that these useful categories can be difficult to define in a uniform way, especially as stomatal regulation alone does not explain leaf water potential maintenance, which depends on other internal and external variables, such as hydraulic conductances throughout the plant, and soil water potential^21,42,43^. Further, the role of these categories in predicting species’ drought tolerance or water use has been questioned^43^. Several have suggested the consideration of alternative approaches for considering overall variation across species in water relations. One alternative recommendation is to consider species on the basis of the response of *g*_s_ to Ψ_leaf_^14^. Indeed, focusing on this response provides insights into processes at a range of scales—the response is directly related to leaf-scale physiology, and easily applied as a component of models to predict plant and ecosystem scale water use^14,44^. A *g*_max_−Ψ_gs50_ trade-off that constrained variation in the response of *g*_s_ to Ψ_leaf_ across species would greatly simplify the consideration of this relationship across species within and across communities and ecosystems. Thus, according to the *g*_max_–Ψ_gs50_ trade-off, many species may be expected to fall along a continuum from high *g*_max_—high Ψ_gs50_ to low *g*_max_—low Ψ_gs50_. Notably, as for other functional trait trade-offs, outliers may be expected, as an important minority of individual species would be expected to depart from this trade-off by evolving independent variation in either trait^45^.

The *g*_max_–Ψ_gs50_ trade-off would likely constrain stomatal behavior in a wide range of natural environments, especially during drought. Among stomatal responses to environmental factors, a quantitatively important role of closure in response to low Ψ_leaf_ in the overall regulation of stomatal conductance is supported by studies of species’ stomatal responses to multiple environmental factors individually and in combination^10,46^, by studies partitioning the role of Ψ_leaf_ in determining *g*_s_ diurnally and during periods of growth^47,48^ and in studies of the role of the response of *g*_s_ to Ψ_leaf_ in predicting ecosystem water use^44^. Notably, the multiple dimensions of stomatal sensitivity to leaf water status present exciting avenues for further research. Species vary not only in the Ψ_leaf_ threshold for a given % closure, as examined in this study, but also in the timing of changes in *g*_s_ in response to changes in water status and multiple other factors^9,25,40,49^. Further studies are needed, for example, to determine whether there is an analogous trade-off between *g*_max_ and the speed of closure during a given level of dehydration.

Future studies are also needed of variation in the response of *g*_s_ to Ψ_leaf_ within and across individuals of a given species, as this response can show strong plasticity. Thus, *g*_max_ and Ψ_gs50_ can change with growing conditions, leaf age, and the degree and duration of water stress regime^11,50,51^. Further research should examine the possibility that the trade-off would apply for given species during progressive or repeated droughts, as would happen if *g*_max_ and Ψ_gs50_ both decline after a drought event due to plasticity^52^.

The *g*_max_–Ψ_gs50_ trade-off has potential implications for ecosystem-level processes. The trade-off would potentially influence species’ distributions along gradients of evapotranspirational demand, as previously shown for safety-efficiency trade-offs in hydraulic conductance^15,23^. Further, the trade-off may scale up to influencing the water use of whole plants and ecosystems. While multiple factors can decouple water use at plant scale from the response of *g*_s_ to Ψ_leaf_, e.g., water storage capacitance, allocation to leaf area relative to sapwood area, and allocation to below versus above-ground biomass^44^, measured and modeled plant and ecosystem water use show strong dependency on the *g*_s_ of the component species^53,54^. Indeed, the response of *g*_s_ to Ψ_leaf_ is fundamental in models for predicting water fluxes of individual plants and ecoregions especially under drought^13,20,38,44,55^. Given its generality, the *g*_max_−Ψ_gs50_ trade-off therefore would have potential applications for prediction of plant water use at a range of scales.

## Methods

### Plant species and growth conditions

We selected 15 morphologically and ecologically diverse tree and shrub species native to California semi-desert, chaparral, coastal scrub, and woodlands (Supplementary Data 2). Plants were cultivated in a greenhouse common garden at the UCLA Plant Growth Center from August 2012 to April 2016^56^. Nine individual seedlings of each species were acquired in 3.8 L pots (Tree of Life Nursery; San Juan Capistrano, CA), and randomized within each of nine blocks containing one individual of each species spread across four greenhouse benches in two greenhouse rooms. Plants were acclimated 12–18 months prior to initial measurements to establish similar external conditions across individuals and species, and to ensure canopies of mature leaves. Plants were carefully monitored for root expansion and repotted when roots filled the pots. Given the species variation in natural history, phenology, and growth rate, 19–38‐L pots were used, as appropriate for each species to minimize stress and accommodate species of different sizes and intrinsic growth rates^57^. Potting soil (18.75% washed plaster sand, 18.75% sandy loam, 37.5% grower grade peat moss, 12.5% horticultural grade perlite, 12.5% coarse vermiculite; Therm-O-Rock West, Inc., Chandler, AZ) was autoclaved prior to use. Plants were irrigated every second day with 200–250 ppm 20:20:20 NPK fertilizer. Daily irradiance ranged up to 1400 μmol m^−2^ s^−1^ (LI-250 light meter; LI-COR Biosciences, Lincoln, NE, USA), while mean minimum, mean and maximum values for temperature were 22.1, 23.9, and 25.2 °C and relative humidity were 47.3%, 60.1%, and 72.8% over the course of our experiments (HOBO Micro Station with Smart Sensors; Onset, Bourne, MA, USA).

Prior to experiments, plants were drought-hardened by watering to field capacity then suspending watering until visible wilting was observed in the morning. A single drought-hardening cycle was used to enable the standardized comparison of plants that had acclimated to strong leaf dehydration, with the recognition that multiple drought cycles may further modify stomatal responses^51^. The initial hardening drought was 1–3 weeks depending on species, and Ψ_leaf_ was measured at mid-day for leaves of three to six individuals per species; species means ranged from −1.1 to −4.3 MPa (Plant Moisture Stress pressure chamber model 1000; PMS Instrument Co., Albany, OR, USA).

### Response of stomatal conductance to leaf dehydration

The response of *g*_*s*_ to dehydration was determined using a refinement of previously used methods applied to excised shoots^15,46^. Three individual plants of each species were selected that had the largest numbers of healthy leaves. Plant shoots with 3–15 leaves (range in length 30–60 cm) were excised from three individual plants per species and rehydrated overnight with cut ends in deionized water and covered with plastic. Mature leaves were sampled from the most recent flushes. At the beginning of the experiment, a water-filled bag was sealed to the cut end of each shoot to maintain full hydration and shoots were acclimated for at least 30 min under high irradiance (>1000 μmol m^−2^ s^−1^; LI-250 light meter; LI-COR Biosciences, Lincoln, NE, USA) and held in frames adaxial side up with fishing line and small pieces of tape at leaf margins on top of a fan. Stomatal conductance was measured on the abaxial surface of given leaves using a porometer (AP-4, Delta-T Devices Ltd, Cambridge, United Kingdom) after which leaves were excised with a razor blade, placed in bags and allowed to equilibrate for at least 30 min before leaf water potential (Ψ_leaf_) was determined (Plant Moisture Stress pressure chamber model 1000; PMS Instrument Co., Albany, OR, USA). A single porometer measurement was taken once stable repeated values were achieved for each leaf before harvesting that leaf for Ψ_leaf_ measurement. Porometry measurements were taken on leaves at intervals ranging from 2 to 60 min as shoots dried, aiming for a range of leaf dehydration. Measurements were made with the bags still attached to the cut ends of shoots, to assess relatively well-hydrated transpiring leaves. Then, the shoot ending with its attached bag was excised using a razor blade, and subsequent measurements were made on remaining leaves as they dehydrated to stomatal closure. We aimed to collect points between maximum opening and full closure across the range of leaf water potentials. All measurements were taken from 0800 to 1400 h. Lab temperature and relative humidity ranged 22.8 ± 0.08 °C and 38.5 ± 0.50%. Notably, our study focused on standardized and controlled measurements of stomatal responses in excised shoots, and their mechanisms. Indeed, most previous studies of stomatal responses to leaf dehydration have focused on excised shoots as tests on a number of species have suggested good agreement with responses measured during the photosynthetic period for whole plants experiencing drought (e.g., refs. ^15,31,46,58^). Yet, some uncertainties remain about scaling shoot scale responses to whole-plants during drought, due to additional influences (e.g., root signals in drying soil)^59^. To further test the potential generality of the trade-off at whole plant scale we also compiled data from previous studies of stomatal responses of leaves on whole plants subjected to drought (see “Compilation and analysis of previous literature.*”*).

### Fitting stomatal responses to leaf water potential

Curves were fitted for the response of stomatal conductance (*g*_s_) to declining leaf water potential (Ψ_leaf_), such that the range of stomatal response characterized was relative to the minimum stomatal conductance. Thus, before curve fitting, the mean minimum epidermal conductance (*g*_min_) for each species, determined for the same experimental plants, was subtracted from each *g*_s_ measurement. For curve-fitting, the datasets for each species were analyzed in two ways. First, all data points were considered for each species’ response of *g*_s_ to Ψ_leaf_ (“all data”). In these responses a number of well-hydrated leaves had closed stomata (“squeeze points”; Supplementary Fig. 4); this closure may represent the effect of the mechanical advantage of epidermal pavement cells or subsidiary cells against guard cells in turgid leaves^29,46^. Further, some leaves showed stomata open when strongly dehydrated beyond the point at which stomata had typically shut, potentially representing re-opening in leaves that lost stomatal control after the epidermis became flaccid^46^ (“re-opening points”; Supplementary Fig. 4). Thus using all data, the responses were not statistically significant for four species. To address these issues, a second dataset (“refined dataset”) was generated excluding the squeeze points, i.e., leaves with *g*_s_ (after subtracting *g*_min_) <50 mol m^−2^ s^−1^ at Ψ_leaf > _−0.5 MPa, and the re-opening points, i.e., *g*_s_ > 50 mol m^−2^ s^−1^ at Ψ_leaf_ > −2.0 MPa; or after the bulk of leaves showed complete stomatal closure. These points constituted 1–14 of 14–77 points per species (3–17%; 8% on average) The responses fitted to this second dataset showed higher goodness of fit (*R*^2^ values), and *P* *<* 0.05 for all species but *Cercocarpus betuloides* (*P* = 0.07; Supplementary Data 4). The parameters calculated from the “all data” and “refined” datasets were highly correlated, and consistent in their ahistorical and evolutionary correlations with other variables (see “*Statistics*” below; Supplementary Data 5 and 6). Thus, the parameters of responses using all data are described in the main text and figures, with the results from both analyses provided in the supplement (Supplementary Data 2).

For each species, we determined the functional response of *g*_s_ to Ψ_leaf_ using maximum likelihood to select among four functions^60^: linear $\left( {g_{\mathrm{s}} = a{\mathrm{\Psi }}_{{\mathrm{leaf}}} + g_{{\mathrm{max}}}} \right)$; sigmoidal $\left[ {g_{\mathrm{s}} = \frac{a}{{1 + {\mathrm{{e}}}^{ - (\frac{{{\mathrm{\Psi }}_{{\mathrm{leaf}} - x_0}}}{b})}}}} \right]$; logistic $\left\{ {g_{\mathrm{s}} = a/\left[ {1 + (\frac{{{\mathrm{\Psi }}_{{\mathrm{leaf}}}}}{{x_0}})^b} \right]} \right\}$; and exponential $\left( {g_{\mathrm{s}} = g_{{\mathrm{max}}} + a{\mathrm{{e}}}^{ - b{\mathrm{\Psi }}_{{\mathrm{leaf}}}}} \right)$. Curves were fitted using the *optim* function in R.2.9.2 (http://www.r-project.org ^61^; our scripts are available on request). The function with the lowest Akaike Information Criterion, corrected for low *n* (AICc) was selected for each data set, with differences > 2 considered as meaningful^61^. From the equations for the selected model, we determined the maximum *g*_s_ for hydrated leaves (*g*_max_), i.e., the *g*_s_ extrapolated to Ψ_leaf _= 0 MPa, and the Ψ_leaf_ corresponding to decline of *g*_s_ by 20%, 50%, and 80% (Ψ_gs20_, Ψ_gs50_, and Ψ_gs80_). We considered various forms of presenting *g*_max_, and used the extrapolated theoretical parameter following previous studies^32,46^. This extrapolated *g*_max_, like other theoretical physiological variables, such as maximum leaf hydraulic conductance, or photosynthetic parameters including the maximum rate of carboxylation (*V*_cmax_), cannot be reached in practice but is useful for generating and testing hypotheses concerning mechanisms or association with other traits^62^. Practically, an extrapolated *g*_max_ was preferred over averaging *g*_s_ values above a threshold Ψ_leaf_, as species differed in the exact Ψ_leaf_ at which *g*_s_ was measured at initial states of dehydration, when *g*_s_ declines steeply in many species. To check that the trade-off did not arise only when using *g*_max_ determined by extrapolation to Ψ_leaf_ = 0 MPa, for the 15 California species we re-analyzed the trend, using *g*_s_ estimated from the selected *g*_s_ versus Ψ_leaf_ functions at Ψ_leaf_ = −0.1 MPa, and calculated Ψ_gs50_ as the Ψ_leaf_ at which *g*_s_ declined by 50%; we found a similar *g*_max_–Ψ_gs50_ trade-off (phylogenetic least squares regression *r* = 0.61; *P* = 0.015; Supplementary Fig. 5). In several species, *g*_s_ begins to decline strongly by Ψ_leaf_ = −0.1 MPa, precluding testing for a safety-efficiency trade-off using lower values of Ψ_leaf_ to estimate “*g*_max_”.

### Compilation and analysis of previous literature

Data were collected from previous studies that reported the responses of *g*_s_ to leaf water potential (Ψ_leaf_), based on searches of Web of Science and Google using search terms “leaf water potential” and “dehydration” or “desiccation”. We compiled all studies that included the response of stomatal conductance to Ψ_leaf_ for two or more species or varieties of a given species, including studies of excised branches, or of potted plants or trees in the field measured for *g*_s_ and Ψ_leaf_ during progressive drought. We included studies with measurements at Ψ_leaf_ > −1.0 MPa and decline of *g*_s_ to 20% of *g*_max_. We found 9 studies of diverse sets of species or varieties, measured with different techniques, and growing conditions (*n* = 2–16; Supplementary Data 2), virtually all of angiosperms, though including one study of two varieties of a fern species^63^. We extracted data points from published figures using ImageJ software version 1.42q. We fitted curves using the same methods as for our experimental plants, though without subtracting *g*_min_ as data were not generally available for the species in the compiled studies.

### Stomatal anatomy

We measured stomatal traits on one leaf from each of three individuals per species. After rehydration, we fixed the leaves in formalin acetic acid (FAA; 48% ethanol: 10% formalin: 5% glacial acetic acid: 37% water). We visualized stomata using nail varnish impressions at the center of the top, middle, and bottom third of the leaf, halfway between the margin and midrib, for the abaxial and adaxial leaf surfaces, using light microscopy. For each image we calculated total stomatal density (*d*) by dividing the number of stomata in the image by the area of the image after subtracting those areas including any blurriness. We calculated mean stomatal areal size (*s*) and width (*W*), and guard cell and stomatal pore lengths (*L* and *p*) for the abaxial surface based on measurements of four stomata selected as nearest to the center of each quadrant of each image. For three species, *Ceanothus spinosus*, *Encelia farinosa*, and *Platanus racemosa*, dense trichomes prevented measurement of stomatal traits. We estimated the theoretical anatomical maximum stomatal conductance (*g*_max,anatomy_^4,62^):

1 $$g_{{\mathrm{max}},{\mathrm{anatomy}}} = \frac{{bmds}}{{s^{0.5}}}$$

In which *b* is a biophysical constant given as $b = \frac{D}{v}$, where *D* represents the diffusivity of water in air (2.82 × 10^−5^ m^2^ s^−1^) and *v* is the molar volume of air (2.24 × 10^−2^ m^3^ mol^−1^); *m* is a factor based on the proportionality of stomatal dimensions ($m = \frac{{\pi c^2}}{{j^{0.5}(4hj + \pi )}}$), with *c* = *p*/*L* and *j* = *W*/*L*. As data were not available for stomatal pore depth, a constant value of 0.5 was assumed for the ratio of stomatal pore depth to width, *h* for the estimation of *g*_max,anatomy_^4,5^. We estimated the stomatal opening ratio as *g*_max_/*g*_max,anatomy_ (*g*_max ratio_, equivalent to the “*a* ratio” in ref. ^5^).

### Pressure–volume curves and leaf structure

Measurements were made of pressure–volume curves and of leaf structure, i.e., leaf dry mass per unit area (LMA), for the study plants^56^. For 6–9 plants per species, 5–6 leaves were measured for leaf water potential and leaf mass during dehydration and from the plotted pressure–volume curves, we determined water potential at full turgor (*π*_o_) and turgor loss point (*π*_tlp_)^59^. For two species with very small leaves and fragile petioles, *C. spinosus* and *Encelia californica*, pressure–volume curves were not constructed.

### Modeling the g_max_–Ψ_gs50_ trade-off at plant scale

To test the hypothesis that a *g*_max_–Ψ_gs50_ trade-off would benefit plant hydraulic design, we used a modeling approach to simulate the consequences for gas exchange and tissue dehydration stresses of variation in *g*_max_ and Ψ_gs50_, and of a trade-off among these variables^38,60^. We implemented a plant hydraulic-stomatal-photosynthetic model based on Darcy’s law, assuming steady-state flow, which simultaneously resolves bulk water potentials (Ψ) and hydraulic conductance (*K*) for each plant organ, given inputs of soil water potential (Ψ_soil_) and VPD and parameters for the response of the hydraulic conductance of whole root, whole stem, and leaf, and of leaf stomatal conductance to water potential within the respective organ. In the model, the volumetric flux of water into each plant component (*F*) is calculated as

2 $$F_{{\mathrm{leaf}}} = \mathop {\int}\nolimits_{{\mathrm{\Psi }}_{{\mathrm{leaf}}}}^{{\mathrm{\Psi }}_{{\mathrm{stem}}}} {K_{{\mathrm{leaf}}}\left( {\mathrm{\Psi }} \right){\mathrm{{d}}}{\mathrm{\Psi }} - g_{\mathrm{s}}({\mathrm{\Psi }}_{{\mathrm{leaf}}}){\mathrm{{VPD}}}}$$

3 $$F_{{\mathrm{stem}}} = \mathop {\smallint }\nolimits_{{\mathrm{\Psi }}_{{\mathrm{stem}}}}^{{\mathrm{\Psi }}_{{\mathrm{root}}}} K_{{\mathrm{stem}}}\left( {\mathrm{\Psi }} \right){\mathrm{{d}}}{\mathrm{\Psi }} - \mathop {\smallint }\nolimits_{{\mathrm{\Psi }}_{{\mathrm{leaf}}}}^{{\mathrm{\Psi }}_{{\mathrm{stem}}}} K_{{\mathrm{{leaf}}}}\left( {\mathrm{\Psi }} \right){\mathrm{{d}}}{\mathrm{\Psi }}$$

4 $$F_{{\mathrm{root}}} = \mathop {\smallint }\nolimits_{{\mathrm{\Psi }}_{{\mathrm{root}}}}^{{\mathrm{\Psi }}_{{\mathrm{soil}}}} K_{{\mathrm{root}}}\left( {\mathrm{\Psi }} \right){\mathrm{{d}}}{\mathrm{\Psi }} - \mathop {\smallint }\nolimits_{{\mathrm{\Psi }}_{{\mathrm{stem}}}}^{{\mathrm{\Psi }}_{{\mathrm{root}}}} K_{{\mathrm{stem}}}\left( {\mathrm{\Psi }} \right){\mathrm{{d}}}{\mathrm{\Psi }}$$

where stomatal conductance is assumed to decline exponentially with Ψ_leaf_. We used an exponential decay function in the modeling, because that response was the most frequently selected by maximum likelihood across species when testing the four functions, i.e., for 7 of 15 species (Supplementary Data 4). In these model simulations, all was kept equal other than *g*_max_ and Ψ_gs50_, including the shape of the response of *g*_s_ to Ψ_leaf_, to assess the consequences of the trade-off; the findings of this modeling exercise would be qualitatively similar using another common stomatal response function. Water transport through the hydraulic system was represented with the Kirchoff transform (i.e., $\mathop {\smallint }_{{\mathrm{\Psi }}_{{\mathrm{leaf}}}}^{{\mathrm{\Psi }}_{{\mathrm{stem}}}} K_{{\mathrm{leaf}}}\left( {\mathrm{\Psi }} \right){\mathrm{{d}}}{\mathrm{\Psi }}$) to account for the non-linearity of the relationship between hydraulic conductance (*K*) and water potential (Ψ)^64^. *K* for each organ was assumed to decline with water potential following a sigmoidal response:

5 $$K = \frac{{K_{{\mathrm{max}}}}}{{1 + {\mathrm{{e}}}^{\alpha \left( {{\mathrm{\Psi }} - {\mathrm{\Psi }}_{50}} \right)}}}$$

where *K*_max_ is the maximum conductance of the plant component, Ψ_50_ is the water potential inducing a 50% decline in conductance, and *α* is a shape parameter. Because water transport is assumed to be at steady-state, the net flux into each component (*F*) is equal to 0. The water potentials of the leaf, stem, and root that satisfied this assumption for given environmental conditions (i.e., vapor pressure deficits (VPDs) and soil water potentials) were then solved using the *fsolve* function in MATLAB (R2016b). Stomatal conductance was then calculated from the *g*_s_ versus Ψ_leaf_ curves. Photosynthesis (*A*_max_) was calculated from *g*_s_ using the equations from the Farquhar model and photosynthetic parameters collected from the literature for *Quercus ilex*, which was selected to represent a typical Mediterranean species^65,66^. Photosynthetic rate was assumed to be light-saturated (i.e, photosynthetically active radiation = 1500 μmol m^−2^ s^−1^), and thus limited only by carboxylation. To derive an expression for *A* as a function of *g*_s,_ Fick’s law of diffusion

6 $$A = u(c_{\mathrm{a}} - c_{\mathrm{i}})$$

where *u* is the stomatal conductance to CO_2_ (*g*_s_/1600, units: mol m^−2^ s^−1^), *c*_a_ is the atmospheric CO_2_ concentration (400 ppm), and *c*_i_ is the intercellular CO_2_ concentration (units: ppm), was substituted into the Farquhar equation for carboxylation-limited photosynthesis

7 $$A = V_{{\mathrm{cmax}}}\frac{{c_{\mathrm{i}} - {\mathrm{\Gamma }}_ \ast }}{{c_{\mathrm{i}} + K_{\mathrm{m}}}}$$

where *V*_cmax_ is the maximum rate of carboxylation (29.1 μmol m^−2^ s^−1^), *K*_m_ is the Rubisco affinity for CO_2_ and O_2_ (550 ppm), and Γ_*_ is the CO_2_ compensation point in the absence of dark respiration (40 μmol m^−2^ s^−1^). This equation was rearranged to produce the expression

8 $$A = a + bu - \sqrt {b^2u^2 + cu + a^2}$$

where these coefficients are equal to

9 $$a = 0.5\left( {V_{{\mathrm{cmax}}} - R} \right)$$

10 $$b = 0.5\left( {c_{\mathrm{a}} + K_{\mathrm{{M}}}} \right)$$

11 $$c = 0.5\left( {R\left( {c_{\mathrm{a}} + K_{\mathrm{M}}} \right) + \,V_{{\mathrm{cmax}}}\left( {K_{\mathrm{M}} - c_{\mathrm{a}} + 2{\mathrm{\Gamma }}^ \ast } \right)} \right)$$

and *R* is the leaf respiration rate (1 μmol m^−2^ s^−1^). The photosynthetic parameters for *Q. ilex* were interpolated from *A*/*C*_i_ curves measured at a constant leaf temperature (30 °C), and leaf temperature was assumed to be constant in the simulations.

We parameterized the model using simulated *g*_s_ versus Ψ_leaf_ responses based on every combination of *g*_max_ values ranging from 100 to 400 mmol m^−2^ s^−1^ in increments of 10 mmol m^−2^ s^−1^ and Ψ_gs50_ values ranging from −0.2 to −3 MPa in increments of 0.2 MPa (Supplementary Data 8). As we did not have data for the hydraulic responses of the dehydration for juveniles of these species, and because we wished to isolate the putative benefit of the *g*_max_–Ψ_gs50_ trade-off, all else being equal, we set all the other parameters of the model at typical values, with leaf, stem, and root hydraulic conductance declining with water potential as a sigmoidal response, with leaf area-normalized maximum hydraulic conductances of leaf, stem, and root systems of 10, 20, and 10 mmol m^−2^ s^−1^ MPa^−1^, respectively, and water potentials at 50% loss of hydraulic conductance of −1, −2 and −1 MPa, respectively. The simulations were performed given soil water potential (Ψ_soil_) of 0 MPa, i.e., high water availability, and −1.2 MPa, i.e., soil drought, under VPD of 1 kPa (0.01 mol mol^−1^). Then, we determined the *g*_s_, *A*, and leaf and stem water potentials.

### Statistics

We analyzed both ahistorical trait correlations and evolutionary correlations among variables to assess both putative physically based mechanisms, as well as evolutionary shifts. We present results of evolutionary correlations in the text, except where specified, and all results in the Supplementary Materials. For ahistorical correlations we used R statistical software to determine Pearson coefficients for untransformed and log-transformed data, to model relationships as either linear or non-linear, i.e., approximately power law^67^. For plotting trait–trait correlations, we used standard major axes to emphasize the structural relationship between two potentially independent variables similar in measurement error (using SMATR^68,69^). A test was made for whether the trade-off, i.e., the *g*_max_ vs. Ψ_gs50_ relationship, varied in slope and/or intercept across studies, for the four studies with ≥6 species (i.e., sufficient species for this test), using log-transformed data, i.e., approximating a power-law relationship, given the nonlinearity of the relationships (using SMATR).

For evolutionary correlations, we applied a phylogenetic generalized least-squares (PGLS) approach to both untransformed and log-transformed data, using a previously published megatree^70^ pruned using the software Phylomatic v3^71^. For analyses based on traits for which values were missing for given species (i.e., for the two species missing *π*_o_ and *π*_tlp_ values, and the three missing stomatal measurements), trees were pruned for the remaining species. For *π*_tlp_ and *π*_o_, which are negative numbers, the values were multiplied by −1. PGLS were calculated using the caper package in R (version 3.4.4)^67^ using models of Brownian motion, Pagel’s lambda, and Ornstein–Uhlenbeck (OU), and the best fit model was selected using the Akaike Information Criterion^72,73^. Reported *r*-values are for the Pagel’s lambda model when the Pagel’s and OU models were equally good fits (difference in AIC score < 2).

### Reporting summary

Further information on research design is available in the Nature Research Reporting Summary linked to this article.

## Supplementary information

Supplementary Information

Description of Additional Supplementary Files

Supplementary Data 1

Supplementary Data 2

Supplementary Data 3

Supplementary Data 4

Supplementary Data 5

Supplementary Data 6

Supplementary Data 7

Supplementary Data 8

Reporting Summary

Source Data

## Data Availability

All data are provided in the Supplementary Data Tables, and the source data underlying Figs. 2–4, and Supplementary Figs. 1–5 are provided as a Source Data file.

## Code availability

Code for all analyses will be made available on request.
