# Supplementary material for: A stomatal safety-efficiency trade-off constrains responses to leaf dehydration
Source: Nat Commun. 2019 Jul 30;10:3398. doi: 10.1038/s41467-019-11006-1 (PMC6667445; doi:10.1038/s41467-019-11006-1)
Supplement: Supplementary file 1 — Supplementary Information [file 41467_2019_11006_MOESM1_ESM.pdf]

Supplementary Information for:

"A stomatal safety-efficiency trade-off constrains responses to leaf dehydration

Christian Henry[1], Grace P. John[1,2], Ruihua Pan[1,3], Megan K. Bartlett[1,4], Leila R. Fletcher[1], Christine Scoffoni[1,5], Lawren Sack[1]*

[1]Department of Ecology and Evolutionary Biology, University of California Los Angeles, 621 Charles E. Young Drive South, Los Angeles, California, 90095, USA

[2]Department of Integrative Biology, University of Texas at Austin, 2415 Speedway, Austin, Texas 78712, USA

[3]School of Ecology and Environment, Inner Mongolia University, 235 University West Road, Hohhot, Inner Mongolia 010021, China

[4]Department of Viticulture and Enology, University of California Davis, One Shields Avenue, Davis, California, 95616, USA

[5]Department of Biological Sciences, California State University, Los Angeles, 5151 State University Drive, Los Angeles, California, 90032, USA

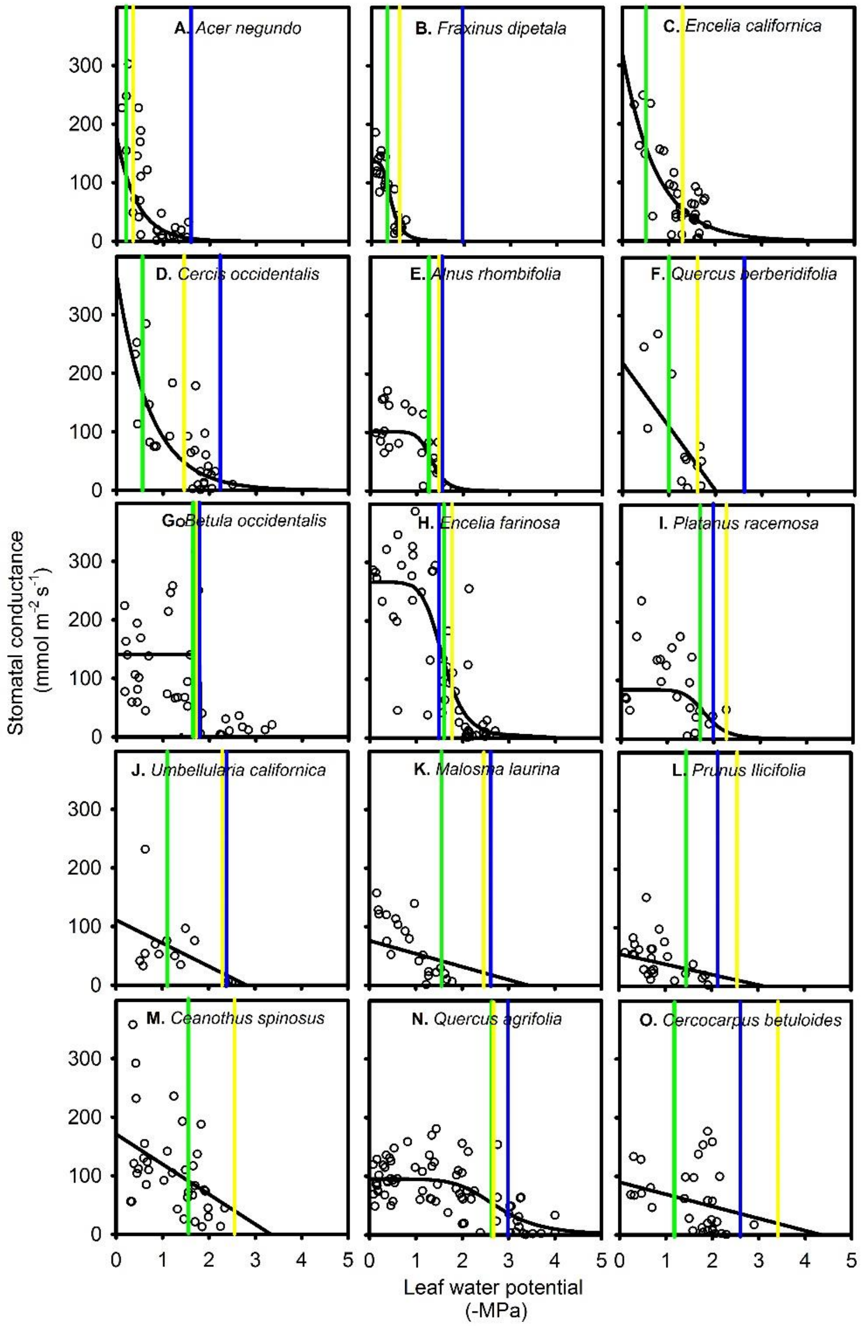

Supplementary Figure 1. Responses of stomatal conductance to declining leaf water potential, for 15 California species grown in a common garden design, ordered in panels (A) to (O) from most to least sensitive in response (number of leaves sampled n = 14-77 across species, depending on the range of the response and the availability of plant material). Curves represent the structural model selected by maximum likelihood; the blue green and yellow lines respectively represent leaf water potentials at turgor loss point, and at 50% and 80% stomatal closure. The "refined" dataset is shown for each species, with the plotted stomatal conductance values representing the measured values minus the minimum epidermal conductance. Source data are provided as a Source Data file.

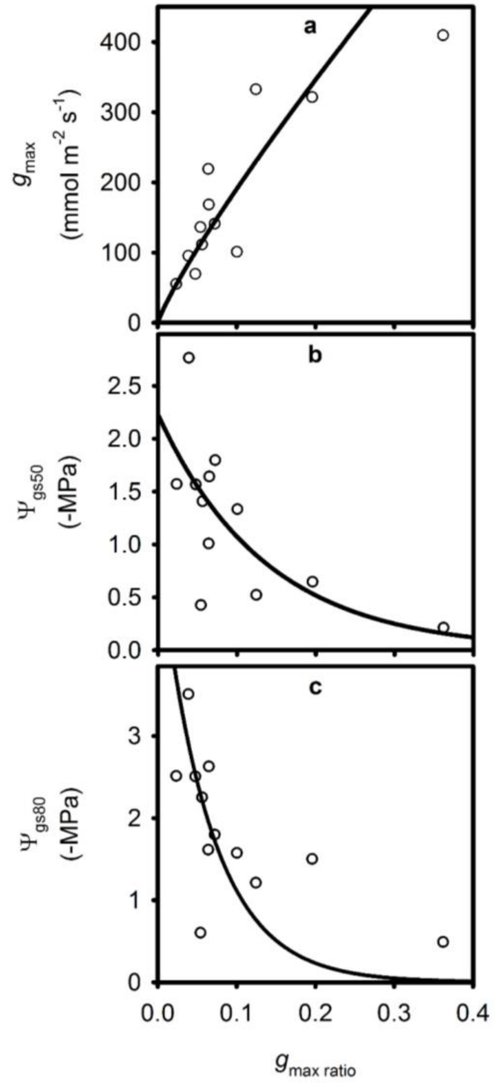

Supplementary Figure 2. Relationships between (A) maximum stomatal conductance (g_{max}), and the leaf water potentials at which stomatal conductance declined by (B) 50% and (C) 80% (Ψ_{gs50} and Ψ_{gs80} respectively) with the stomatal opening ratio ($g_{max\ ratio} = g_{max}$ / maximum anatomical stomatal conductance) for California species grown in a common garden design. Lines are standard major log-transformed data, i.e., for power law fits. Phylogenetic least squares regression r values were 0.63, 0.62, and 0.86 (P-values 0.03, 0.03 and 0.0004) for panels A, B and C respectively. The g_{max}, Ψ_{gs50} and Ψ_{gs80} were derived from fitted curves (Supplementary Figure 1). Source data are provided as a Source Data file.

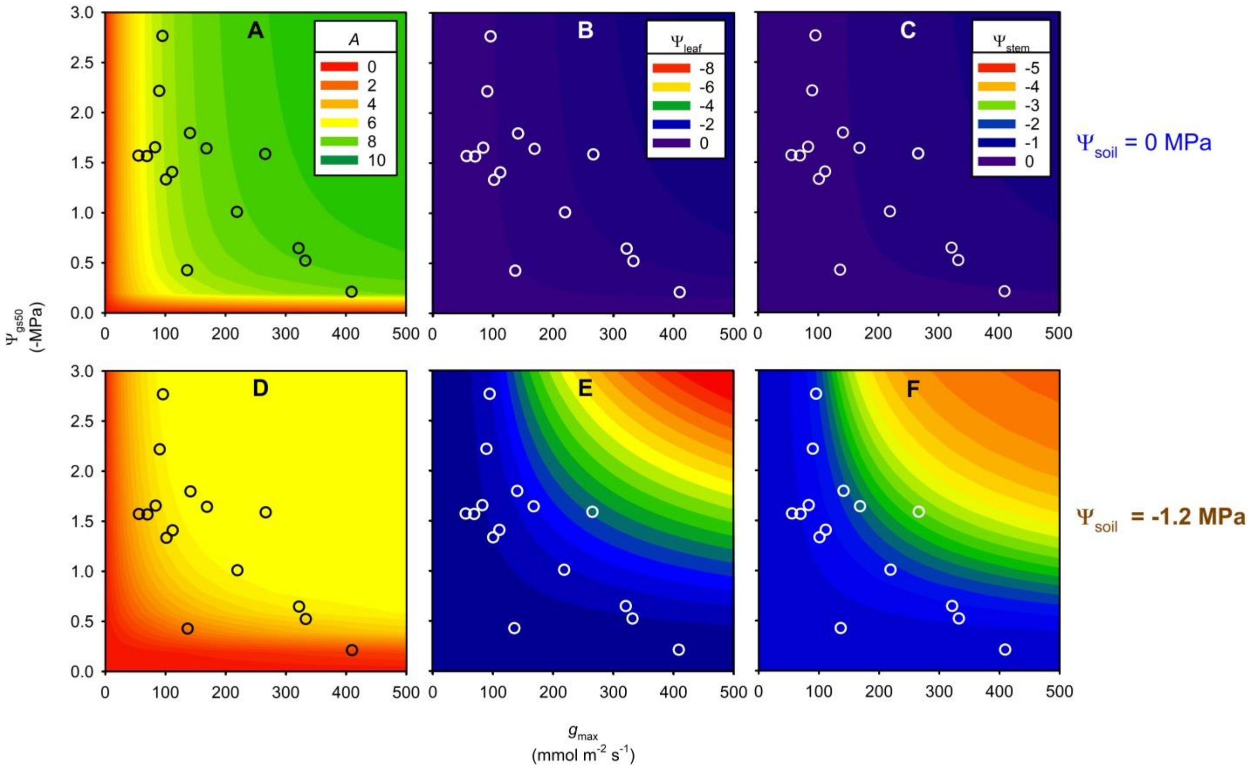

Supplementary Figure 3. Testing for a benefit in plant hydraulic design of a stomatal efficiency-safety trade-off, using a plant hydraulic-stomatal model. (A and D) Modelled light saturated photosynthetic rate (*A*; units: μmol m^{-2} s^{-1}) for simulated species with varying combinations of maximum stomatal conductance (g_{max}) and sensitivity to stomatal closure (Ψ_{gs50}) under high water availability, i.e., soil water (Ψ_{soil}) of 0 MPa or under low water availability, i.e., Ψ_{soil} of -1.2 MPa, showed that higher *A* is achieved by species with higher g_{max} or less sensitive stomatal closure (low Ψ_{gs50}); (B, C, E and F) modelled leaf and stem water potentials (Ψ_{leaf} and Ψ_{stem} respectively; units: MPa) showed that steep declines in Ψ_{leaf} and Ψ_{stem} occurred for species with higher g_{max} and insensitive stomata (i.e., very negative Ψ_{gs50}), with both Ψ_{leaf} and Ψ_{stem} showing extreme values under drought, whereas species with high Ψ_{gs50} were protected from dehydration stress. The plotted points represent the 15 California species, for which the observed g_{max} vs Ψ_{gs50} trade-off positioned these species in the sweet spot, maintaining *A* under high water availability and avoiding hydraulic damage during drought. See Methods for model description and parameterization. Source data are provided as a Source Data file.

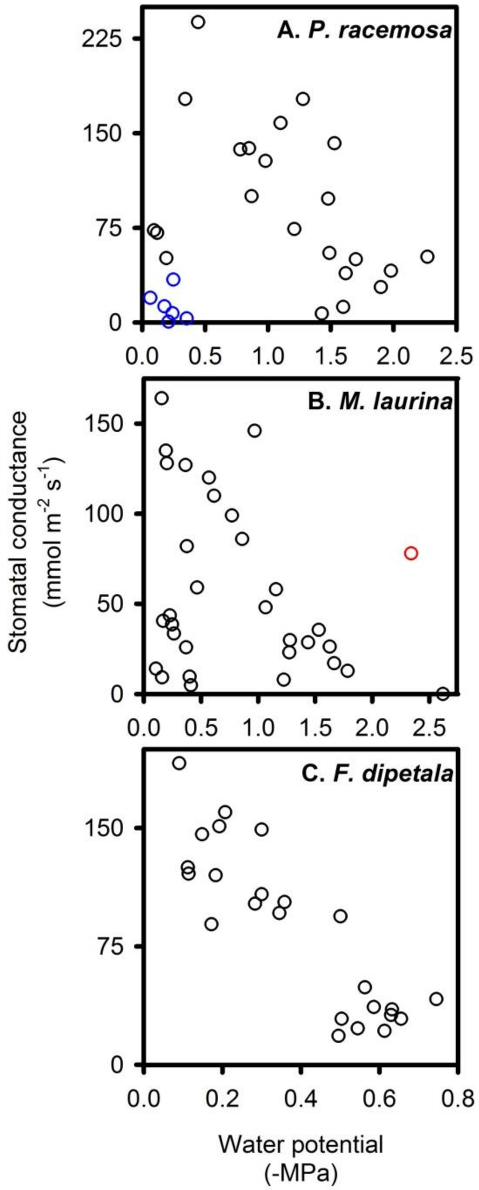

Supplementary Figure 4. Response of stomatal conductance to decline in leaf water potential for three species, showing infrequent "squeeze" points, i.e., closed stomata at high water potentials (panel A, blue points), or a "re-opening" point, i.e., stomata open at very low water potentials (panel B, red point), and a species' response that lacked either (panel C). The plotted stomatal conductance values represent the measured values minus the minimum epidermal conductance. Source data are provided as a Source Data file.

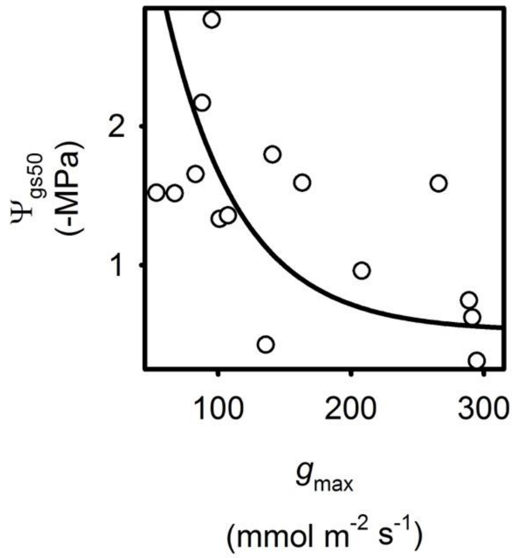

Supplementary Figure 5. The stomatal safety-efficiency trade-off, i.e., the relationship of leaf water potential at 50% stomatal closure during dehydration (Ψ_{gs50}) to maximum stomatal conductance (g_{max}) for 15 California species grown in a common garden design, using an alternative calculation of g_{max} and Ψ_{gs50}. Here, g_{max} was estimated as stomatal conductance (g_s) estimated from the selected g_s versus leaf water potential (Ψ_{leaf}) functions at Ψ_{leaf} = -0.1 MPa, and Ψ_{gs50} was estimated as the Ψ_{leaf} at which that g_s declined by 50%; we found a similar g_{max} - Ψ_{gs50} trade-off (phylogenetic r = 0.61; P = 0.015). The fitted line is a standard major axis fitted to log-transformed data, i.e., for a power law fit. Source data are provided as a Source Data file.
