# Supplementary material for: A stomatal safety-efficiency trade-off constrains responses to leaf dehydration
Source: Nat Commun. 2019 Jul 30;10:3398. doi: 10.1038/s41467-019-11006-1 (PMC6667445; doi:10.1038/s41467-019-11006-1)
Supplement: Supplementary file 2 — Description of Additional Supplementary Files [file 41467_2019_11006_MOESM2_ESM.pdf]

Description of Additional Supplementary Files

File Name: Supplementary Data 1
Description: Species of 15 woody California species grown in a common garden design, with family, native ecosystem, life form, leaf habit and mean and standard error values for stomatal response parameters, and for epidermal anatomy, leaf structure, and pressure volume parameters.

File Name: Supplementary Data 2
Description: Nine previous studies of stomatal responses to declining leaf water potential, and our analyses for the presence of a trade-off between maximum stomatal conductance (gmax) and the leaf water potentials at which stomatal conductance declined by 20, 50 and 80%.

File Name: Supplementary Data 3
Description: Stomatal responses to leaf dehydration for 15 woody California species grown in a common garden design.

File Name: Supplementary Data 4
Description: Parameters of models fitted to stomatal responses to leaf water potential in 15 California woody species grown in a common garden design.

File Name: Supplementary Data 5
Description: Correlation matrix for 15 California species grown in a common garden design, for stomatal response parameters, epidermal anatomy, leaf structure, and pressure volume parameters.

File Name: Supplementary Data 6
Description: Phylogenetic generalized least squares (PGLS) regression tests of trait pairs for 15 diverse California species grown in a common garden design.

File Name: Supplementary Data 7
Description: Parameters of models fitted to stomatal responses to leaf water potential for the data of previous studies.

File Name: Supplementary Data 8
Description: Model simulations to test the hypothesis that a stomatal safety efficiency tradeoff, i.e., between maximum stomatal conductance (gmax) and the leaf water potential at 50% stomatal closure (Ψgs50), would benefit whole plant hydraulic design.
