# Supplementary material for: A stomatal safety-efficiency trade-off constrains responses to leaf dehydration
Source: Nat Commun. 2019 Jul 30;10:3398. doi: 10.1038/s41467-019-11006-1 (PMC6667445; doi:10.1038/s41467-019-11006-1)
Supplement: Supplementary file 11 — Reporting Summary [file 41467_2019_11006_MOESM11_ESM.pdf]

nature research

Corresponding author(s): Lawren Sack

Last updated by author(s): May 23, 2019

Reporting Summary

Nature Research wishes to improve the reproducibility of the work that we publish. This form provides structure for consistency and transparency in reporting. For further information on Nature Research policies, see Authors & Referees and the Editorial Policy Checklist.

Statistics

For all statistical analyses, confirm that the following items are present in the figure legend, table legend, main text, or Methods section.

n/a	Confirmed	
☐	☒	The exact sample size (*n*) for each experimental group/condition, given as a discrete number and unit of measurement
☐	☒	A statement on whether measurements were taken from distinct samples or whether the same sample was measured repeatedly
☐	☒	The statistical test(s) used AND whether they are one- or two-sided<br>*Only common tests should be described solely by name; describe more complex techniques in the Methods section.*
☒	☐	A description of all covariates tested
☒	☐	A description of any assumptions or corrections, such as tests of normality and adjustment for multiple comparisons
☐	☒	A full description of the statistical parameters including central tendency (e.g. means) or other basic estimates (e.g. regression coefficient) AND variation (e.g. standard deviation) or associated estimates of uncertainty (e.g. confidence intervals)
☐	☒	For null hypothesis testing, the test statistic (e.g. *F*, *t*, *r*) with confidence intervals, effect sizes, degrees of freedom and *P* value noted<br>*Give P values as exact values whenever suitable.*
☒	☐	For Bayesian analysis, information on the choice of priors and Markov chain Monte Carlo settings
☒	☐	For hierarchical and complex designs, identification of the appropriate level for tests and full reporting of outcomes
☒	☐	Estimates of effect sizes (e.g. Cohen's *d*, Pearson's *r*), indicating how they were calculated

Our web collection on statistics for biologists contains articles on many of the points above.

Software and code

Policy information about availability of computer code

Data collection	ImageJ
Data analysis	R language, Minitab 18

For manuscripts utilizing custom algorithms or software that are central to the research but not yet described in published literature, software must be made available to editors/reviewers. We strongly encourage code deposition in a community repository (e.g. GitHub). See the Nature Research guidelines for submitting code & software for further information.

Data

Policy information about availability of data

All manuscripts must include a data availability statement. This statement should provide the following information, where applicable:

- Accession codes, unique identifiers, or web links for publicly available datasets
- A list of figures that have associated raw data
- A description of any restrictions on data availability

All data are provided in the Supplementary Data Tables, and the source data underlying Figures 2-4, and Supplementary Figures 1-5 are provided as a Source Data file.

Field-specific reporting

Please select the one below that is the best fit for your research. If you are not sure, read the appropriate sections before making your selection.

☐ Life sciences ☐ Behavioural & social sciences ☒ Ecological, evolutionary & environmental sciences

Ecological, evolutionary & environmental sciences study design

All studies must disclose on these points even when the disclosure is negative.

Study description	Stomata, the microvalves on leaf surfaces, exert major influences across scales, from plant growth and productivity to global carbon and water cycling . Stomatal opening enables leaf photosynthesis, and plant growth and water use, whereas plant survival of drought depends on stomatal closure. Here we report that stomatal function is constrained by a safety-efficiency trade-off, such that species with greater stomatal conductance under high water availability (gmax) show greater sensitivity to closure during leaf dehydration, i.e., a higher leaf water potential at which stomatal conductance is reduced by 50% (Ψgs50). The gmax - Ψgs50 trade-off and its mechanistic basis is supported by experiments on leaves of California woody species, and in analyses of previous studies of the responses of diverse flowering plant species around the world. Linking the two fundamental key roles of stomata—the enabling of gas exchange, and the first defense against drought— this trade-off constrains the rates of water use and the drought sensitivity of leaves, with potential impacts on ecosystems.
Research sample	We selected 15 morphologically and ecologically diverse tree and shrub species native to California semi-desert, chaparral, coastal scrub and woodlands. Plants were cultivated in a greenhouse common garden at the UCLA Plant Growth Center from August 2012 to April 2016.
Sampling strategy	Nine individual seedlings of each species were acquired in 3.8 liter pots (Tree of Life Nursery; San Juan Capistrano, CA), and randomized within each of nine blocks containing one individual of each species spread across four greenhouse benches in two greenhouse rooms.
Data collection	The response of stomata to dehydration was assessed for each species by the research team. Measurements were also made of stomatal anatomy, osmotic potentials and leaf structure.
Timing and spatial scale	Measurements were made from 2016-2017
Data exclusions	No data were excluded from analyses
Reproducibility	Analyses of previously published data were made to test the generality of our experimental findings.
Randomization	Nine individual seedlings of each species were acquired in 3.8 liter pots (Tree of Life Nursery; San Juan Capistrano, CA), and randomized within each of nine blocks containing one individual of each species spread across four greenhouse benches in two greenhouse rooms.
Blinding	Blinding was not relevant to this study of plant physiological responses.

Did the study involve field work? ☐ Yes ☒ No

Reporting for specific materials, systems and methods

We require information from authors about some types of materials, experimental systems and methods used in many studies. Here, indicate whether each material, system or method listed is relevant to your study. If you are not sure if a list item applies to your research, read the appropriate section before selecting a response.

Materials & experimental systems

n/a	Involved in the study	
☒	☐	Antibodies
☒	☐	Eukaryotic cell lines
☒	☐	Palaeontology
☒	☐	Animals and other organisms
☒	☐	Human research participants
☒	☐	Clinical data

Methods

n/a	Involved in the study	
☒	☐	ChIP-seq
☒	☐	Flow cytometry
☒	☐	MRI-based neuroimaging
